# Electrical Property Characterization of Neural Stem Cells in Differentiation

**DOI:** 10.1371/journal.pone.0158044

**Published:** 2016-06-24

**Authors:** Yang Zhao, Qingxi Liu, He Sun, Deyong Chen, Zhaohui Li, Beiyuan Fan, Julian George, Chengcheng Xue, Zhanfeng Cui, Junbo Wang, Jian Chen

**Affiliations:** 1 State Key Laboratory of Transducer Technology, Institute of Electronics, Chinese Academy of Sciences, Beijing, P.R. China; 2 Tianjin Weikai Bioeng Ltd., Tianjin, P.R. China; 3 Tianjin University of Science & Technology, Tianjin, P.R. China; 4 Institute of Biomedical Engineering, Department of Engineering Science, Oxford University, Oxford, United Kingdom; Temple University School of Medicine, UNITED STATES

## Abstract

Electrical property characterization of stem cells could be utilized as a potential label-free biophysical approach to evaluate the differentiation process. However, there has been a lack of technology or tools that can quantify the intrinsic cellular electrical markers (e.g., specific membrane capacitance (C_specific membrane_) and cytoplasm conductivity (σ_cytoplasm_)) for a large amount of stem cells or differentiated cells. In this paper, a microfluidic platform enabling the high-throughput quantification of C_specific membrane_ and σ_cytoplasm_ from hundreds of single neural stem cells undergoing differentiation was developed to explore the feasibility to characterize the neural stem cell differentiation process without biochemical staining. Experimental quantification using biochemical markers (e.g., Nestin, Tubulin and GFAP) of neural stem cells confirmed the initiation of the differentiation process featured with gradual loss in cellular stemness and increased cell markers for neurons and glial cells. The recorded electrical properties of neural stem cells undergoing differentiation showed distinctive and unique patterns: 1) in the suspension culture before inducing differentiation, a large distribution and difference in σ_cytoplasm_ among individual neural stem cells was noticed, which indicated heterogeneity that may result from the nature of suspension culture of neurospheres; and 2) during the differentiation in adhering monolayer culture, significant changes and a large difference in C_specific membrane_ were located indicating different expressions of membrane proteins during the differentiation process, and a small distribution difference in σ_cytoplasm_ was less significant that indicated the relatively consistent properties of cytoplasm during the culture. In summary, significant differences in C_specific membrane_ and σ_cytoplasm_ were observed during the neural stem cell differentiation process, which may potentially be used as label-free biophysical markers to monitor this process.

## Introduction

Electrical properties of single cells have been regarded as label-free and cost-effective biophysical parameters for cell status evaluation and type classification without the requirement of extensive antibodies [1–4]. As one of key biophysical markers, they have been used to classify cell types including tumour cells [5–11], stem cells [12–15], red blood cells [16, 17] and white blood cells [18–20].

In the field of electrical property characterization of stem cells, dielectrophoresis is the golden approach where the number of cells attached to dielectrophoretic electrodes at a group of frequencies is recorded to form “dielectrophoretic collection spectrum”, which can be further translated to intrinsic cellular electrical properties [21]. Based on this technique, the differentiation processes of neural stem cells [14, 15] and mesenchymal stem cells [12] were monitored, respectively. Although powerful, this technique can only provide electrical properties based on batch testing and cannot quantify electrical properties at the single cell level, incapable of addressing the issue of heterogeneity in the cellular differentiation process.

With the development of microfluidics, due to its dimensional comparison with biological cells (1–100 μm [22, 23]), it has been used for single-cell analysis including the quantification of both biochemical and biomechanical properties [24, 25]. In the field of cellular electrical property characterization, microfluidic impedance flow cytometry has been proposed as a bioengineering tool capable of high-throughput single-cell property characterization [4, 26, 27]. Song et al. provided a microfluidic coulter counter to monitor the differentiation process of embryonic stem cells based on impedance data 50 kHz, 250 kHz, 500 kHz and 1 MHz [13]. However, in this study, only raw impedance depending on experimental conditions and cellular sizes were obtained, and due to the lack of electrical models, these raw data cannot be translated to intrinsic cellular electrical markers and cannot be effectively compared.

Recently, we proposed a microfluidic impedance flow cytometry to aspirate single cells through a constriction channel with a cross-section area smaller than cells under measurement. An electrical model was developed to translate raw impedance obtained for the cellular travelling process in the constriction channel to size-independent intrinsic electrical parameters of C_specific membrane_ (cellular membrane capacitance per area) and σ_cytoplasm_ (cellular cytoplasm conductance per length)[5, 28]. Based on these platforms, intrinsic cellular electrical properties of hundreds of single cells were obtained, enabling the classification of 1) cell types with and without fixation and surface staining [29]; 2) tumour cells and their counterparts with single oncogenes under regulation [11].

In this study, leveraging the developed microfluidic platform, the electrical properties of the differentiation process of neural stem cells were quantified with unique patterns recorded. Before the initiation of the differentiation process, a large distribution difference in σ_cytoplasm_ among individual neural stem cells was noticed while during the differentiation process, a large distribution difference in C_specific membrane_ was located, indicating different expressions of membrane proteins as a sign of heterogeneity in the differentiation process.

## Materials and Methods

### Materials

Materials used for cell culture and differentiation regulations include Dulbecco’s Modified Eagle’s Medium (DMEM, Gibco, Carlsbad, CA, USA), F12 (Gibco, Carlsbad, CA, USA), RPMI1640 (Gibco, Carlsbad, CA, USA), Minimum essential medium (MEM, Sigma-Aldrich, Irine, United Kingdom), epidermal growth factor (EGF, Gibco, Carlsbad, CA, USA), basic fibroblast growth factor (bFGF, Gibco, Carlsbad, CA, USA), B27 (Gibco, Carlsbad, CA, USA), N2 (Gibco, Carlsbad, CA, USA), Glutamax (Gibco, Carlsbad, CA, USA), Heparin (Gibco, Carlsbad, CA, USA), HEPES (Gibco, Carlsbad, CA, USA), D-Glucose (Gibco, Carlsbad, CA, USA), Laminin (Gibco, Carlsbad, CA, USA), fetal bovine serum (FBS, Gibco, Carlsbad, CA, USA), live/dead viability-cytotoxicity kit (Invitrogen, Carlsbad, CA, USA), penicillin-streptomycin (Pen/Strep, Gibco, Carlsbad, CA, USA), accutase^TM^ (Sigma, Saint Louis, MO, USA), NaHCO_3_ (Sigma, Saint Louis, MO, USA), NaCl (Sigma-Aldrich) and all-trans-retinoic acid (ATRA, Sigma-Aldrich). Materials required for device fabrication included SU-8 photoresist (MicroChem Corp., Newton, MA, USA) and 184 silicone elastomer (Dow Corning Corporation, Midland, MI, USA).

### Isolation, Culture and Differentiation of Neural Stem Cells

The experimental work was approved by the Committee on the Ethics of Animal Experiments of Tianijn Weikai Bioeng Ltd., following the guidelines used in the UK, and the Committee on the Ethics of Animal Experiments of Tianjin International Joint Academy for Biotechnology and Medicine (Certificate Number: 20120225), where parts of the experimental procedures were carried out.

The rats were kept in OptiMice IVC cages (size: 470 mm x 300 mm x 150 mm) in special cleanrooms with filtered incoming air, under regular cycles of 12/12 hour light/dark, constant room temperature of 20–26°C, and relative humidity of 40%-70%. The rats were fed with 5 gram food and 8–11 ml water (100 gram body weight/24 hours) every day.

The isolation of rat neural stem cells was carried out strictly following the established procedure [30] with every effort to minimize animal suffering. Briefly the rat neural stem cells were obtained through dissociation of embryonic hippocampus tissues from Sprague-Dawley (SD) rats with 14 days of pregnancy, sacrificed by cervical dislocation (The Animal Experiment Center of Academy of Military Medical Sciences, Beijing, China [license number SCXK-(military) 2012–0004]). The tissue samples were minced and incubated in Accutase^TM^ at 37°C for 10 min. The harvested cells were cultured in the suspension in serum-free primary culture medium, consisting of DMEM/RPMI1640/F12 mixture (1:1:1, v/v/v) supplemented with 10 ng/ml of EGF, 20 ng/ml of bFGF, 2 mM Glutamax, 1% B27, 5 mmol/L HEPES and 5 mg/mL Heparin at a density of 1x10^5^ cells/ml in T25 cm^2^ cell culture flasks (37°C and 5% CO_2_). When neurospheres reached the diameter of 150 μm, cell digestion was conducted to form 1x10^5^ cells for qPCR and 1x10^5^ cells for bioelectrical property characterization, which is labeled as Day 0.

The differentiation of neural stem cells was described in detail in [31] and briefly, on Day 1, the neural stem cells were plated at a density of 1x10^5^ cells/cm^2^ in 24 Well Plates with RPMI1640 plating medium consisting of 10% FBS, 1% MEM and 1% Pen/Strep. From Day 2, RPMI1640 differentiation media containing 10% FBS, 1% MEM, 1% S Pen/Strep and 10 μmol/L ATRA was used to replace the RPMI1640 plating medium and refreshed every 2–3 Days. Before each experiment, the same digestion process was used for both biochemical and bioelectrical property characterization.

### Biochemical Characterization of Neural Stem Cells

Total RNA was extracted from the cells using the TRIzol reagent according to the manufacturer’s instructions and stored at -80°C. The first-strand cDNA synthesis reaction was performed with 0.1 μg pure total RNA using Transcripor First Strand cDNA Synthesis Kit. Real-Time PCR reactions were performed using the SYBR® GreenER™ qPCR SuperMix kit with the ABI 7500 Fast Real-Time PCR apparatus (USA). Two pairs of primer were designed to amplify each gene: GFAP (Forward: 5’-AGGCTGCTGGAGCAAGACAA-3’, Reverse: 5’-GCCTTAGTGGCCATTCCAGGTA-3’, product 147 bp), Tubulin (Forward: 5’-CAGATGCTGGCCATTCAGAGTAAG-3’, Reverse: 5’-TGTTGCCGATGAAGGTGGAC-3’, product 127 bp), Nestin (Forward: 5’-CTGTGGCTCACCTGCTGGAA-3’, Reverse: 5’- CAGATAAATGCTGCCCTCAGCTC-3’, product 142 bp) and β-actin (Forward: 5’- TGGCACCCAGCACAATGAA-3’, Reverse: 5’- CTAAGTCATAGTCCGCCTAGAAGCA-3’, product 186 bp), all of which were synthesized by Invitrogen.

### Bioelectrical Characterization of Neural Stem Cells

The characterization of single-cell electrical properties based on the developed microfluidic platform was described in detail in [5, 28]. Briefly, the two-layer polydimethylsiloxane (PDMS) device (constriction channel cross-section area smaller than cells) was replicated from a double-layer SU-8 mold based on conventional lithography. The first layer of SU-8 5 was used to form the constriction channel and the second layer of SU-8 25 was used to form the cell loading channel. PDMS prepolymer and curing agent were mixed, degassed, poured on channel masters, and baked in an oven. PDMS channels were then peeled from the SU-8 masters with reservoir holes punched through and bonded to a glass slide.

In experiments, cell samples at a concentration of 1 million cells per ml were pipetted to the entrance of the cell loading channel of the microfluidic device, where a negative pressure of 1 kPa was applied to aspirate cells continuously through the constriction channel with two-frequency impedance data (1 kHz + 100 kHz) and images recorded. The dimensions of the constriction channels used in this study were 6 x 6 μm^2^ for the neural stem cells of day 0 and day 1 and 8 x 8 μm^2^ for the neural stem cells of day 3 and day 7 since during the differentiation process, a gradual increase in cellular sizes was noticed. Note that the collected electrical markers of C_specific membrane_ and σ_cytoplasm_ were independent from both the channel cross-sectional areas and cell sizes.

The detailed procedures for data analysis were described previously [5, 28], and summarized as follows. Raw impedance data at 1 kHz were used to evaluate the sealing properties of deformed cells with constriction channel walls, while raw impedance data at 100 kHz were used to quantify equivalent cellular membrane capacitance and cytoplasm resistance. By combining cell elongation length during its traveling process within the constriction channel based on image processing, equivalent membrane capacitance and cytoplasm resistance were further translated to C_specific membrane_ and σ_cytoplasm_.

### Statistics

In each group, the measurement results were expressed by means ± standard deviations. All biochemical characterization data on Day 1, Day 3 and Day 7 were normalized to data obtained on Day 0. ANOVA (S-N-K method, coding in Excel) was used for multiple-group comparisons where values of P <0.05 (*) and P <0.01 (**) were considered statistical significance and high statistical significance, respectively.

## Results and Discussion

In this study, we explored the feasibility of using electrical parameters including C_specific membrane_ (specific membrane capacitance, as an indicator of cellular membrane properties) and σ_cytoplasm_ (cytoplasm conductivity, as an indicator of cellular cytoplasm properties) to monitor the differentiation of neural stem cells. The conventional characterization of stem cell differentiation using RT-PCR was conducted as the confirmation of neural stem cells in states of differentiation. Neural stem cells *in vitro* are able to generate neurospheres that exhibit heterogenetic properties containing neuronal and glial progenitors, in addition to neural stem cells at different states of differentiation [32]. Nestin (stemness marker), Tubulin (neuronal marker) and GFAP (glial cell marker) were set as confirmations of the differentiation of neural stem cells.

**Fig 1:**
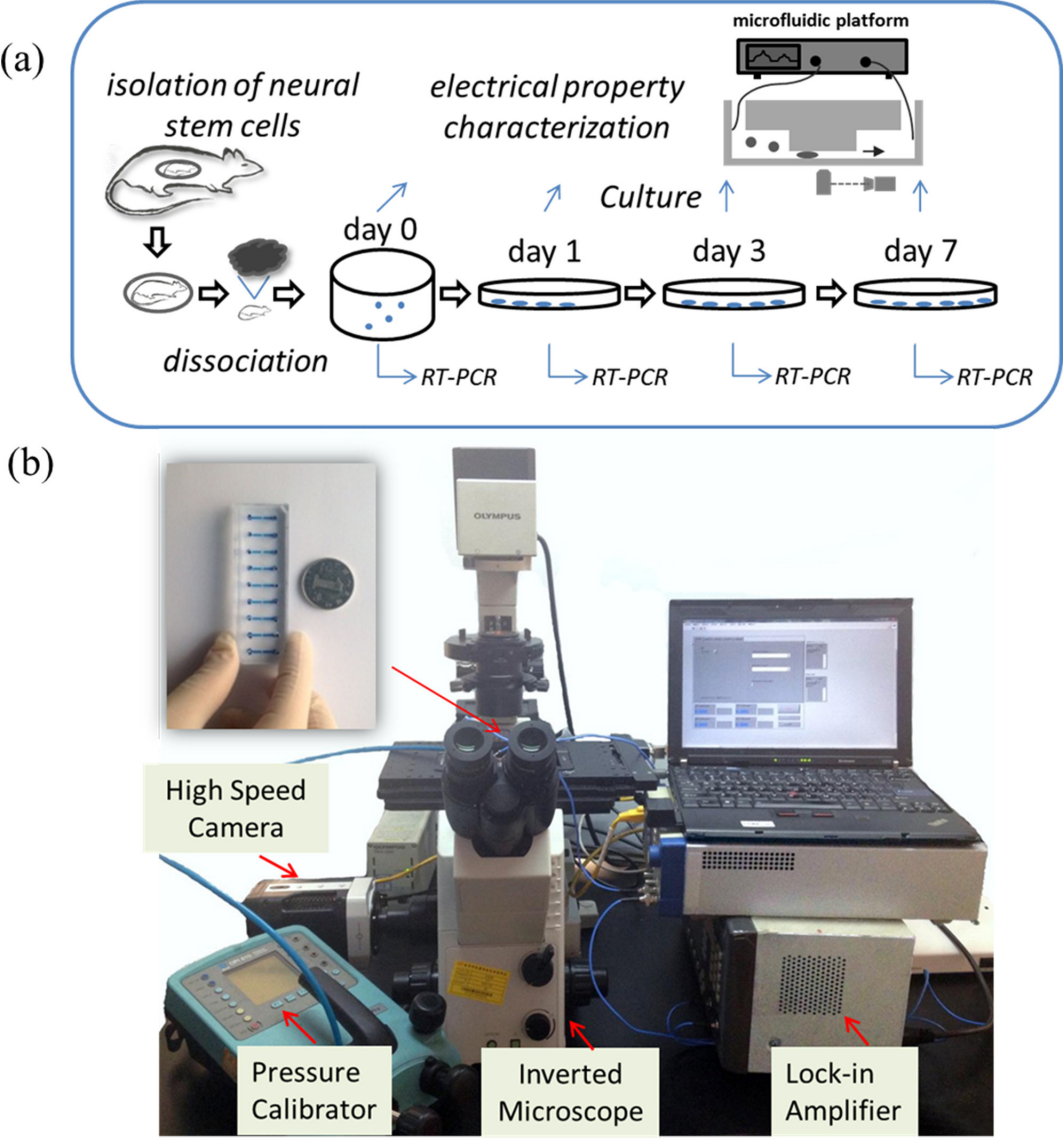
(a) Schematic of the experimental design composed of stem cell isolation and differentiation with biochemical and bioelectrical parameters quantified. The cell culture steps include isolation of neural stem cells from rats, suspension culture of neural stem cells (Day 0) and differentiation of neural stems in the adhesive culture (Day 1, Day 3, and Day 7). On Day 0, Day 1, Day 3 and Day 7, two portions of neural stem cells were collected for the characterization of cellular biochemical parameters based on RT-PCR and bioelectrical parameters based on the home-developed impedance microfluidic flow cytometry, respectively. (b) Experimental setup of the microfluidic system for continuous characterization of C_specific membrane_ and σ_cytoplasm_ of single cells in suspension where cells are aspirated continuously through the constriction channel with impedance data at 1 kHz and 100 kHz and cell elongation length measured by a lock-in amplifier and an inverted microscope simultaneously.

The experimental design and bioelectrical characterisation set-up were shown in Fig 1. Neural stem cells, initially cultured in the suspension state (Day 0), were transferred to adhesive culture to start the differentiation process (Day 1, Day 3 and Day 7). On Day 0, Day 1, Day 3 and Day 7, two portions of neural stem cells were collected for the characterization of cellular biochemical markers (e.g., Nestin, Tubulin and GFAP) based on RT-PCR and bioelectrical parameters (e.g., C_specific membrane_ and σ_cytoplasm_) based on the microfluidic platform, respectively.

**Fig 2:**
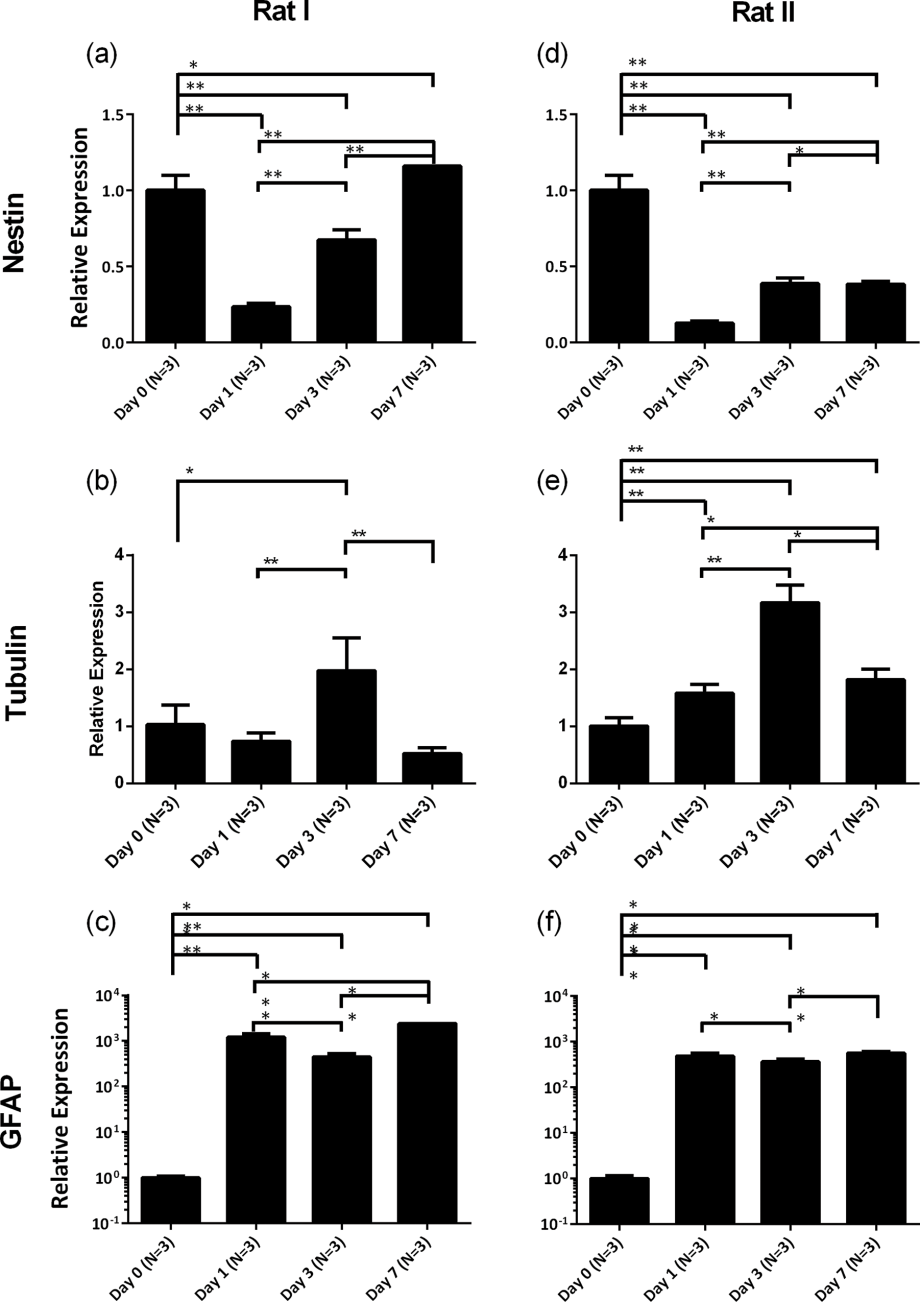
**The mRNA changes of neuronal specific markers of Nestin ((a) for rat I and (d) for rat II), Tubulin ((b) for rat I and (e) for rat II), and GFAP ((c) for rat I and (f) for rat II), during the differentiation process of neural stem cells.** In each group, the measurement results were expressed by averages and standard deviations. ANOVA (S-N-K method, coding in Excel) was used for multiple-group comparisons where values of P <0.05 (*) and P <0.01 (**) were considered statistical significance and high statistical significance, respectively.

Fig 2 recorded the RT-PCR results of three biochemical markers of neural stem cells during the differentiation process (data collection from two rats). The relative expression of Nestin as the marker of stemness was quantified as 1.00±0.10 on Day 0, 0.23±0.02 on Day 1, 0.67±0.07 on Day 3, 1.16±0.0 on Day 7 for rat I (see Fig 2(A)), and 1.00±0.10 on Day 0, 0.13±0.01 on Day 1, 0.39±0.04 on Day 3, 0.38±0.02 on Day 7 for rat II. Compared to the expression of Nestin on Day 0, decreased expressions were quantified for both rats, indicating the initiation of the differentiation process and the gradual loss of cellular stemness (see Fig 2(A) and 2(D)). Note that for the first week of differentiation, the stemness of neural stem cells was retained to a certain degree and thus a fluctuation in the expression of Nestin was observed, which should decrease steadily after day 7. To the contrast, the population of neurons increased steadily with culture while the population of glial cells increased rapidly during the early differentiation process. The relative expression peaks of Tubulin were located on Day 3, which were characterized as 1.98±0.57 for rat I and 3.17±0.31 for rat II, while the expressions on Day 1 (0.74±0.14 for rat I and 1.58±0.15 for rat II) and Day 7 (0.52±0.10 for rat I and 1.82±0.18 for rat II) were relatively low (see Fig 2(B) and 2(E)). In this study, the use of serum in the differentiation medium has a side effect on the differentiation of neural stem cells to neurons which may lead to regulated expression of Tubulin on day 7. It is believed that the removal of serum in the differentiation medium can lead to increased expression of Tubulin as more neural stem cells may be differentiated towards neurons.

The expression of GFAP was significantly high in the early differentiation process with fluctuated patterns. The relative expression peaks were observed on Day 1 (1204.17±234.58 for rat I and 483.97±70.91 for rat II) and Day 7 (2421.13±0.00 for rat I and 563.18±55.12 for rat II) while the expression on Day 3 was relatively low (444.74±86.64 for rat I and 363.49±53.26 for rat II) (see Fig 2(C) and 2(F)). This might reflect that glial cells possess an intrinsic tendency to proliferate during differentiation, particularly when exposed to serum-containing medium. However, glial cell overgrowth can be restrained in the subsequent growth when medium shelf to serum-free neuronal maintenance medium for further culture.

**Fig 3:**
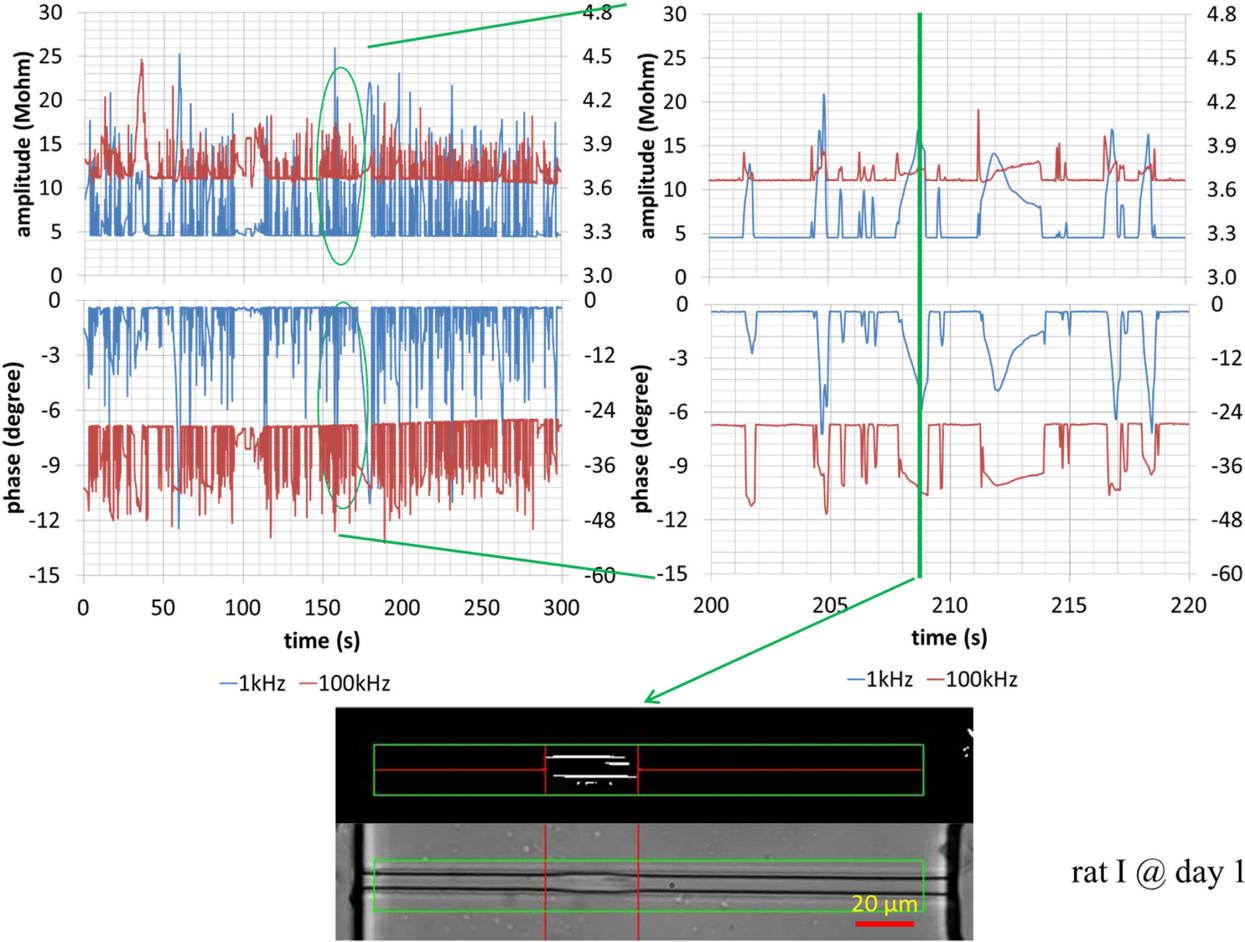
Typical impedance amplitude and phase measurement at 1 kHz and 100 kHz, simultaneously as well as cellular images squeezing through the constriction channels for a single neural stem cell for rat 1 on Day 1. During the cell squeezing process, there is an amplitude increase and phase decrease for data at both 1 kHz and 100 kHz. Basal impedance amplitudes (no cell entry) at 1 kHz were lower than the values at 100 kHz and there were higher impedance amplitude increases during the cellular squeezing process at 1 kHz than those of 100 kHz. As to the phase data, basal phase values (no cell entry) at 1 kHz were almost zero degree, which were higher than the values at 100 kHz. There were higher phase changes during the cellular squeezing process at 100 kHz than 1 kHz.

Typical impedance amplitude and phase measurement at 1 kHz and 100 kHz, simultaneously as well as images of cellular squeezing through the constriction channels for a neural stem cells of rat 1 on Day 1 were shown in Fig 3. During the cell squeezing process, there was an amplitude increase and a phase decrease for impedance data at both 1 kHz and 100 kHz. Basal impedance amplitudes (no cell entry) at 1 kHz were lower than the values at 100 kHz and there were higher impedance amplitude increases during the cellular squeezing process at 1 kHz than those of 100 kHz. As to the phase data, basal phase values (no cell entry) at 1 kHz were almost zero degree, which were higher than the values at 100 kHz. There were higher phase changes during the cellular squeezing process at 100 kHz than 1 kHz. The similar trend was observed from other testing time point and another rat as well.

**Fig 4:**
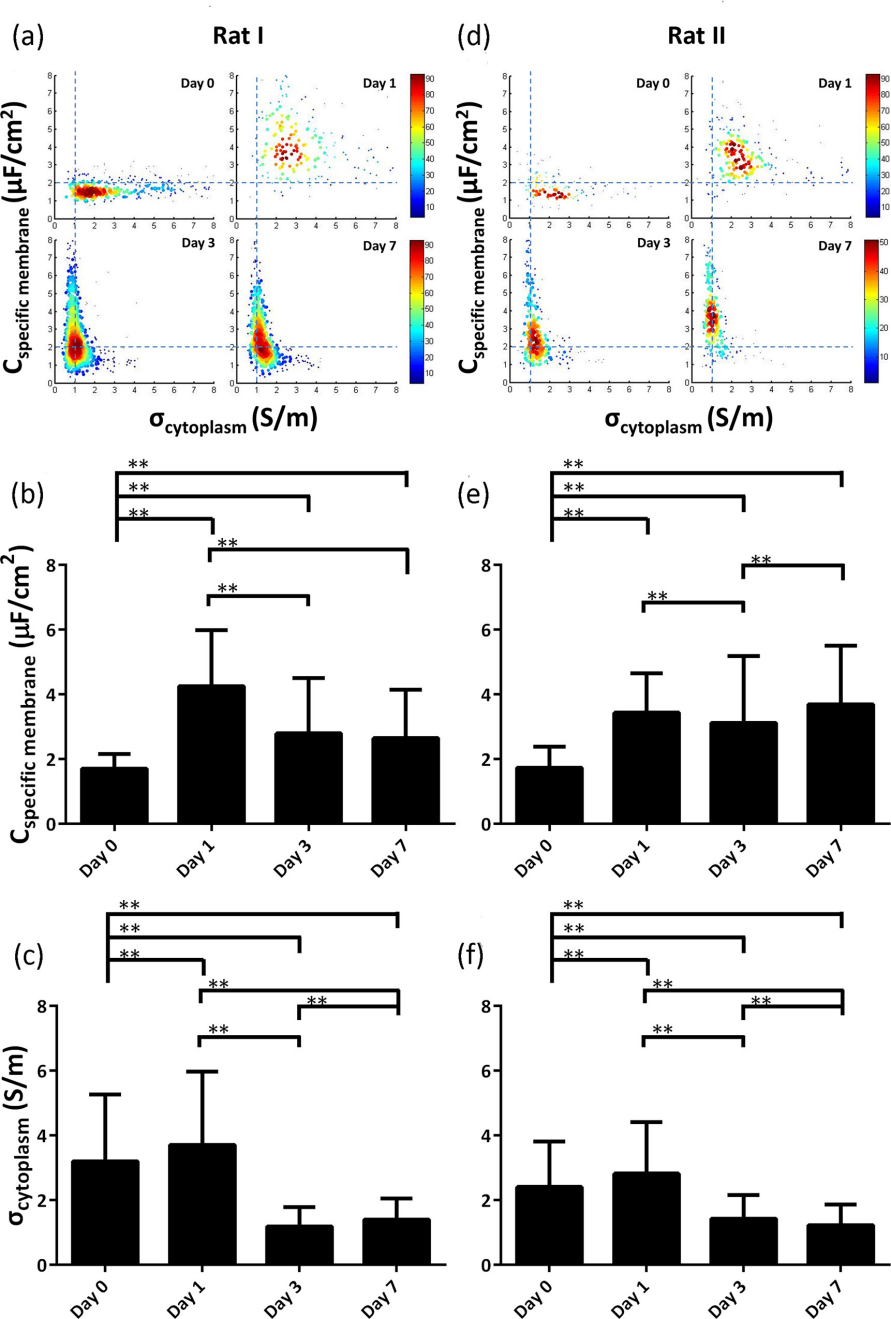
**C_specific membrane_ and σ_cytoplasm_ of neural stem cells during the differentiation process as a function of time ((a) for rat I and (d) for rat II).** Before the initiation of differentiation, a large distribution difference in σ_cytoplasm_ among individual neural stem cells was noticed, indicating heterogeneity in cytoplasm which may result from the nature of suspension culture (Day 0). After seeding cells in microplates to start the differentiation process, large distribution differences in both C_specific membrane_ and σ_cytoplasm_ were observed (Day 1). As time goes by, only a large distribution difference in C_specific membrane_ was observed indicating expression variations in membrane proteins for differentiated neural stem cells while a small distribution was located for σ_cytoplasm_ which may result from the adhesive culture in the differentiation process. Meanwhile, means and standard deviations of C_specific membrane_ ((b) for Rat I and (e) for Rat II) and σ_cytoplasm_ ((c) for Rat I and (f) for Rat II) were expressed and statistical analysis were conducted using ANOVA (S-N-K method, coding in Excel) for multiple-group comparisons where values of P <0.05 (*) and P <0.01 (**) were considered statistical significance and high statistical significance, respectively.

Fig 4 showed C_specific membrane_ and σ_cytoplasm_ of neural stem cells isolated from two rats on Day 0, Day 1, Day 3 and Day 7, recording significant differences in cellular electrical properties during the differentiation process. C_specific membrane_ and σ_cytoplasm_ on Day 0 were quantified as 1.71±0.45 μF/cm^2^ and 3.21±2.05 S/m (n_cell_ = 343) for rat I and 1.74±0.66 μF/cm^2^ and 2.41±1.40 S/m (n_cell_ = 129) for rat II. As shown in Fig 4, a large distribution difference in σ_cytoplasm_ among individual neural stem cells was noticed, which may result from the nature of suspension culture where cell clusters were formed, leading to cellular heterogeneity in cytoplasm.

As to the electrical properties of neural stem cells on Day 1, C_specific membrane_ and σ_cytoplasm_ were quantified as 4.26±1.73 μF/cm^2^ and 3.71±2.26 S/m (n_cell_ = 205) for rat I and 3.44±1.22 μF/cm^2^ and 2.83±1.59 S/m (n_cell_ = 217) for rat II. Compared to the case of Day 0, a large distribution difference in C_specific membrane_ was located among individual cells, which indicates heterogeneous expression of membrane proteins, resulting from the initiation of cellular differentiation.

On Day 3, a significant different pattern of the scatter plot was obtained in comparison to the electrical data collected on Day 1, where C_specific membrane_ and σ_cytoplasm_ were quantified as 2.80±1.71 μF/cm^2^ and 1.19±0.59 S/m for rat I (n_cell_ = 624) and 3.12±2.07 μF/cm^2^ and 1.43±0.73 S/m for rat II (n_cell_ = 257). Compared to the data collected on Day 1, only a large distribution in C_specific membrane_ was observed, indicating the existence of different expressions of membrane proteins during the differentiation process. Meanwhile, a small distribution in σ_cytoplasm_ among individual neural stem cells was observed on Day 3 which may result from the 2D monolayer adhesive culture in the differentiation process. Similar data was collected on Day 7 where a large distribution in C_specific membrane_ and a small distribution in σ_cytoplasm_ were quantified as 2.65±1.50 μF/cm^2^ and 1.40±0.65 S/m for rat I (n_cell_ = 473) and 3.70±1.81 μF/cm^2^ and 1.22±0.64 S/m for rat II (n_cell_ = 216).

## Conclusion

During the differentiation of neural stem cells, biochemical indicators (e.g., GFAP, Tubulin and Nestin) were assayed and bioelectrical properties (e.g., C_specific membrane_ and σ_cytoplasm_) were quantified at several time points. The results of biochemical assays confirmed the differentiation process featured with a graduate decrease in cell stemness. The monitoring of the two indicative bioelectrical parameters, C_specific membrane_ and σ_cytoplasm_, of the cells showed their changes during the neural stem cell differentiation. The observed patterns can be summarized as the following, 1) for the cells from the neurosphere suspension culture before the initiation of the differentiation process, there exists a large distribution difference in σ_cytoplasm_ among individual neural stem cells, which may result from the nature of suspension culture of neurospheres; and 2) for the cells at different stages of differentiation, there is a large distribution difference in C_specific membrane_ among individual neural stem cells, indicating different and changing expressions of membrane proteins. The results indicate that it is feasible to use bioelectrical properties of neural stem cells to monitor their differentiation, although future work using more homogeneous stem cell populations to quantify and to link the changes of the parameter changes with degree of differentiation is required.
